# Determinants of Peri-Procedural Mechanical Complications During Peripheral Endovascular Revascularization: Insights from Single-Center Experience

**DOI:** 10.3390/life16020213

**Published:** 2026-01-28

**Authors:** Thierry Unterseeh, Livio D’Angelo, Youcef Lounes, Francesca Sanguineti, Antoinette Neylon, Hakim Benamer, Benjamin Honton, Antoine Sauguet, Antonella Millin, Julius Jelisejevas, Giacomo Maria Cioffi, Stephane Cook, Mario Togni, Neila Sayah, Pietro Laforgia, Nicolas Amabile, Thomas Hovasse, Philippe Garot, Mariama Akodad, Stephane Champagne, Ioannis Skalidis

**Affiliations:** 1 Institut Cardiovasculaire Paris-Sud, Hôpital Jacques Cartier, 91300 Massy, France; t.unterseeh@angio-icps.com (T.U.); l.dangelo@angio-icps.com (L.D.);; 2 Department of Cardiology, Clinique Pasteur, 31300 Toulouse, France; 3 Department of Cardiology, HFR—Fribourg Cantonal Hospital and University, 1700 Fribourg, Switzerland

**Keywords:** peripheral artery disease, endovascular intervention, procedural complications

## Abstract

**Background:** Peripheral endovascular intervention is the preferred revascularization strategy for patients with chronic lower-limb ischemia. Although generally safe, peri-procedural mechanical complications may occur and are influenced by both lesion complexity and procedural strategy. Data identifying determinants of such complications in routine clinical practice remain limited. **Methods:** We performed a retrospective single-center analysis of consecutive patients undergoing peripheral endovascular intervention for chronic lower-limb ischemia between 2010 and 2023. The primary endpoint was the occurrence of peri-procedural mechanical complications, defined as mechanical adverse events occurring during or immediately following the index intervention and directly related to catheter manipulation, device deployment, or vascular access. Lesion- and procedure-related predictors were evaluated using multivariable logistic regression analysis. **Results:** A total of 283 index procedures were included. Peri-procedural mechanical complications occurred in 9 procedures (3.2%), with arterial dissection being the most frequent event (2.1%). No cases of peri-procedural bleeding, distal embolization, or emergent surgical conversion were observed. In multivariable analysis, chronic total occlusion (adjusted odds ratio [aOR] 1.89, 95% confidence interval [CI] 1.14–3.11; *p* = 0.014), moderate-to-severe arterial calcification (aOR 1.74, 95% CI 1.03–2.93; *p* = 0.039), introducer sheath size ≥7 French (aOR 2.08, 95% CI 1.21–3.57; *p* = 0.007), and ≥3 vascular access attempts (aOR 1.67, 95% CI 1.00–2.81; *p* = 0.048) were associated with increased risk of peri-procedural mechanical complications in adjusted analyses. **Conclusions:** In this real-world institutional registry, peri-procedural mechanical complications during peripheral endovascular intervention were uncommon. Lesion complexity and procedural factors, rather than access route or device type, were the primary determinants of mechanical risk. These findings highlight the importance of careful lesion assessment and procedural planning to optimize peri-procedural safety in routine practice.

## 1. Introduction

Peripheral artery disease (PAD) remains a major global health concern, affecting more than 236 million individuals worldwide, with increasing prevalence among aging populations and those with diabetes, chronic kidney disease, and tobacco exposure [1]. Among patients with chronic limb-threatening ischemia (CLTI), which is the most severe form of PAD, the risks of cardiovascular mortality, limb loss, and repeated hospitalization remain particularly high [2,3].

Endovascular therapy (EVT) has become the preferred revascularization strategy for infrainguinal PAD owing to its minimally invasive nature, rapid recovery, and expanding device landscape [3]. Despite these advantages, peri-procedural mechanical complications, such as arterial dissection, perforation, and access site mechanical injury, may still occur, particularly in anatomically complex lesions [4].

Lesion morphology plays a critical role in procedural safety. Chronic total occlusions (CTOs) and severe arterial calcification are consistently associated with increased technical difficulty, prolonged procedure duration, and higher complication rates [4,5]. In the BIOLUX P-III registry, CTOs and long lesions were independently associated with increased late lumen loss and target lesion revascularization, underscoring the importance of anatomical complexity not only for long-term outcomes but also for procedural safety during endovascular intervention [6]. Beyond anatomy, procedural factors such as access strategy, sheath size, and the number of puncture attempts have also emerged as important contributors to procedural risk. For instance, the DEFINITIVE AR study demonstrated that procedural complexity influenced complication rates even when advanced vessel preparation devices were used [7].

Contemporary large registries, including the Vascular Quality Initiative (VQI), IN.PACT Global, DEFINITIVE LE, and post hoc analyses of VOYAGER PAD, have shown that complication rates and practice patterns vary widely across institutions, driven by differences in operator experience, lesion characteristics, and device availability [8,9,10,11]. However, these multicenter datasets predominantly represent high-volume centers or device-specific cohorts and often lack granular integration of both anatomical and procedural variables. As a result, real-world evidence from medium-volume centers, where resources, operator caseload, and procedural strategies differ substantially, remains underrepresented. Importantly, these registries often focus on clinical outcomes or device-specific endpoints, with less emphasis on peri-procedural mechanical complications occurring during the index intervention.

These limitations highlight a persistent gap in the understanding of how combined lesion- and procedure-related characteristics influence the risk of peri-procedural mechanical complications in unselected PAD populations treated in routine practice. Quality improvement analyses from single institutions may therefore provide valuable insights for benchmarking performance, identifying modifiable procedural factors, and informing local clinical protocols.

Accordingly, this study aimed to identify lesion- and procedure-related predictors of peri-procedural mechanical complications occurring during the index peripheral endovascular intervention, using a 10-year real-world institutional registry of patients with chronic lower-limb ischemia.

## 2. Materials and Methods

### 2.1. Study Design and Setting

This retrospective, single-center observational cohort study was conducted at the Institut Cardiovasculaire Paris Sud, Hôpital Privé Claude Galien/Ramsey Santé, Quincy-sous-Sénart, France, and included patients who underwent peripheral endovascular revascularization for chronic lower limb ischemia between January 2010 and October 2023. The registry was conducted in accordance with the Declaration of Helsinki and was approved by the institutional ethics committee. All patients provided informed consent for anonymized research use of their data.

### 2.2. Study Population

Eligible patients were adults (≥18 years) treated with endovascular intervention for chronic lower-limb ischemia, including de novo or restenotic lesions involving the iliac, femoropopliteal, or below-the-knee arterial segments. Chronic ischemia was defined based on clinical symptoms and imaging findings consistent with Rutherford classification categories ≥2.

Patients were excluded if they underwent intervention for acute limb ischemia, hybrid surgical–endovascular procedures, isolated diagnostic angiography without intervention, or if key procedural data were missing or incorrectly recorded. Because the primary endpoint was peri-procedural, post-discharge follow-up duration was not a criterion for inclusion. For patients undergoing multiple interventions during the study period, only the index procedure was considered for analysis to preserve statistical independence. This approach was chosen to avoid clustering effects and overrepresentation of patients undergoing multiple interventions, particularly given the low event rate. For each index procedure, the dominant treated lesion was selected for analysis; thus, all anatomical and procedural variables correspond to a single lesion–procedure pair per patient.

### 2.3. Data Collection and Variables

Demographic characteristics, cardiovascular risk factors, comorbidities, lesion morphology, and procedural details were prospectively recorded in the institutional registry at the time of intervention. Data completeness was routinely audited as part of the registry’s quality assurance process.

### 2.4. Lesion Characteristics

Anatomical variables included treated arterial territory, presence of chronic total occlusion (CTO), lesion length, reference vessel diameter, TASC II classification, and angiographic calcification severity. Calcification severity was assessed qualitatively by the operating physician using standard angiographic criteria and categorized as none/mild or moderate-to-severe.

### 2.5. Procedural Characteristics

Procedural variables included vascular access route (femoral, radial, or brachial), introducer sheath size, number of vascular access attempts, balloon strategy (pre-dilatation and/or post-dilatation), and stent implantation strategy (direct stenting or staged ballooning). Stent type was categorized as bare-metal, drug-eluting, or covered stent.

Vascular access was obtained per standard practice at the operator’s discretion. The choice of access route was based on lesion location and extent, anticipated device profile and sheath-size requirements, iliofemoral anatomy and access feasibility, and overall procedural strategy. The use of ultrasound guidance for vascular access evolved over the study period and was heterogeneous among operators and across years; however, ultrasound-guided access was not captured as a dedicated registry variable and therefore could not be quantified or analyzed in relation to peri-procedural mechanical complications.

Procedures were performed within a dedicated peripheral endovascular program, with the majority of interventions carried out by four main operators over the study period.

### 2.6. Outcomes and Definitions

The primary endpoint was the occurrence of any peri-procedural mechanical complication during the index endovascular intervention. Peri-procedural mechanical complications were defined as mechanical adverse events occurring during or immediately following the procedure, directly attributable to catheter manipulation, device deployment, or vascular access, and recorded prospectively in the procedural registry. These complications included arterial dissection, arterial perforation, access site mechanical complications, device-related mechanical failure, and other procedure-related mechanical events explicitly documented by the operator. The primary endpoint was analyzed at the index procedure level and treated as a binary outcome (presence or absence of ≥1 peri-procedural mechanical complication). Clinical adverse events occurring after the procedure (e.g., bleeding, ischemia, stroke, myocardial infarction, restenosis, ulceration, or death) were not included in the primary endpoint, as they represent distinct clinical outcomes with different timing and pathophysiology. Access site hematomas and pseudoaneurysms typically occur in the post-procedural period and were therefore not included in the primary peri-procedural mechanical complication endpoint.

Complications were operator-reported at the time of the procedure in predefined registry fields and were not externally adjudicated; no independent events committee or angiographic core laboratory review was performed. The registry captures peri-procedural mechanical complications as categorical events and does not include formal grading of angiographic severity (e.g., dissection grade) or systematic classification according to treatment requirement. Accordingly, the present analysis assesses the presence/absence of operator-documented peri-procedural mechanical complications. Minor, non-flow-limiting angiographic dissections that did not require additional treatment may not have been consistently recorded and may therefore be under-captured.

### 2.7. Data Collection and Variables

Baseline demographic characteristics, comorbidities, lesion morphology, and procedural parameters were prospectively entered into the institutional database at the time of intervention and updated during follow-up. Anatomical variables included lesion territory, presence of chronic total occlusion (CTO), TASC II classification, lesion length, reference vessel diameter, and calcification severity, assessed using standardized qualitative angiographic criteria aligned with VIVA/VQI morphology descriptors. Procedural variables included vascular access route, introducer sheath size, number of puncture attempts, balloon strategy (pre-/post-dilatation), stent type, and use of adjunctive devices (e.g., atherectomy, lithotripsy). Missing data were minimal across variables. Calcification severity was missing in six cases (2.1%), whereas all other variables included in the multivariable model were complete. Given the low level of missingness, a complete-case approach was used for the regression analyses.

### 2.8. Statistical Analysis

Continuous variables are summarized as mean ± standard deviation or median with interquartile range, as appropriate based on data distribution. Categorical variables are presented as counts and percentages. Group comparisons were performed using the chi-square test or Fisher’s exact test for categorical variables and Student’s *t*-test or Mann–Whitney U test for continuous variables, as appropriate. Univariable logistic regression analyses were conducted to identify potential predictors of peri-procedural mechanical complications. Variables associated with the endpoint at a *p*-value < 0.10 in univariable analysis, as well as variables considered clinically relevant, were entered into a multivariable logistic regression model using a backward elimination strategy. The primary multivariable analysis was performed at the index procedure level using a complete-case (listwise deletion) approach, resulting in a final analytic sample of 277 procedures with complete data for all covariates. To preserve analytical coherence and avoid mixing hierarchical data levels, only anatomical and procedural variables related to the index procedure were included in the primary model; patient-level variables were not incorporated. All statistical analyses were performed using R statistical software (4.R Foundation for Statistical Computing, Vienna, Austria). A two-sided *p*-value < 0.05 was considered statistically significant.

### 2.9. Ethical Considerations

The study protocol was reviewed and approved by the local institutional ethics committee. Written informed consent was obtained from all participants for the use of anonymized clinical data for research purposes, in accordance with the Declaration of Helsinki and local regulatory requirements.

## 3. Results

### 3.1. Study Population

Between January 2010 and October 2023, 285 patients underwent peripheral endovascular intervention for chronic lower-limb ischemia at our institution. Two patients were excluded because of erroneous registry inclusion, leaving 283 patients who constituted the final study population. Only the index procedure was analyzed for patients who underwent multiple interventions during the study period. Baseline clinical and lesion characteristics are summarized in Table 1. The mean age of the cohort was 67.5 ± 11.2 years, and 79.2% of patients were male. Cardiovascular risk factors were common, including hypertension (68.9%), dyslipidemia (60.1%), diabetes mellitus (28.6%), and current or former smoking (70.3%). Chronic total occlusion (CTO) was present in 38.2% of treated lesions, and moderate-to-severe angiographic calcification was observed in 48.8%.

### 3.2. Procedural Characteristics

Procedural characteristics are detailed in Table 2. Most interventions involved the iliac (47.7%) or femoropopliteal (51.2%) arterial segments. Femoral access was used in 71.0% of procedures, radial access in 23.3%, and brachial access in 5.7%. An introducer sheath size ≥7 French was required in 6.4% of procedures, and three or more vascular access attempts were recorded in 24.7%. Balloon pre-dilatation and post-dilatation were performed in 56.9% and 48.4% of procedures, respectively, and direct stenting was used in 38.5%. Bare-metal stents were the most frequently implanted devices (62.4%), followed by drug-eluting stents (25.0%) and covered stents (12.6%). Hemostasis was achieved using vascular closure devices or manual compression. Closure strategies included ProGlide in 80 cases (28.3%), Angio-Seal in 56 (19.8%), Femoseal in 33 (11.7%), TR Band in 51 (18.0%), and manual compression in 58 cases (20.5%); closure failure was rare (2 cases, 0.7%). (Table 3).

### 3.3. Incidence and Distribution of Peri-Procedural Mechanical Complications

In multivariable analysis (Table 4), chronic total occlusion was associated after limited multivariable adjustment with an increased risk of peri-procedural mechanical complications (adjusted odds ratio [aOR] 1.89, 95% confidence interval [CI] 1.14–3.11; *p* = 0.014). Moderate-to-severe arterial calcification was also associated with a higher complication risk (aOR 1.74, 95% CI 1.03–2.93; *p* = 0.039). Procedural factors demonstrated a significant association with mechanical risk. Use of an introducer sheath size ≥7 French was associated with more than a twofold increase in peri-procedural complications (aOR 2.08, 95% CI 1.21–3.57; *p* = 0.007). Similarly, procedures requiring three or more vascular access attempts were associated with an increased risk of mechanical complications (aOR 1.67, 95% CI 1.00–2.81; *p* = 0.048). Lesion location, vascular access route, and stent type were not associated with peri-procedural mechanical complications after multivariable adjustment.

### 3.4. Predictors of Peri-Procedural Mechanical Complications

Multivariable logistic regression identified four predictors of peri-procedural mechanical complications. Chronic total occlusion was associated with an adjusted odds ratio (aOR) of 1.89 (95% confidence interval [CI]: 1.14–3.11; *p* = 0.014). Moderate-to-severe arterial calcification was also associated with increased risk (aOR: 1.74; 95% CI: 1.03–2.93; *p* = 0.039). The use of introducer sheaths ≥7 French conferred a higher risk of peri-procedural mechanical complications (aOR: 2.08; 95% CI: 1.21–3.57; *p* = 0.007). Procedures requiring three or more vascular access attempts were similarly associated with increased risk (aOR: 1.67; 95% CI: 1.00–2.81; *p* = 0.048). Lesion location (iliac vs. femoropopliteal), vascular access route, and stent type were not associated with peri-procedural mechanical complications after multivariable adjustment.

## 4. Discussion

In this single-center, real-world registry of patients undergoing peripheral endovascular intervention for chronic lower-limb ischemia, peri-procedural mechanical complications were infrequent, occurring in only 3.2% of index procedures. Despite the overall low complication rate, several lesion- and procedure-related factors were associated in adjusted analyses with increased mechanical risk, underscoring the importance of lesion complexity and procedural execution in determining peri-procedural safety.

The overall incidence of peri-procedural mechanical complications observed in this cohort is consistent with contemporary reports from routine endovascular practice, particularly in mixed lesion populations treated outside of highly selected device trials. Arterial dissection was the most frequent complication, followed by isolated cases of perforation and other mechanical events, whereas no peri-procedural bleeding, distal embolization, or emergent surgical conversion was observed. These findings suggest that, in experienced hands, peripheral endovascular intervention can be performed with a low rate of acute mechanical failure, even in anatomically complex disease.

Importantly, the present analysis focused strictly on mechanical complications occurring during or immediately following the index procedure, deliberately excluding delayed clinical adverse events such as restenosis, ischemia, stroke, or bleeding. This distinction is critical, as peri-procedural mechanical complications reflect technical execution and device–vessel interaction, whereas post-procedural clinical events are influenced by a broader array of biological, pharmacological, and disease-progression factors. By isolating mechanical events, the present study provides a clearer assessment of procedural risk attributable to lesion anatomy and operator-dependent factors.

Chronic total occlusion and moderate-to-severe arterial calcification emerged as predictors of peri-procedural mechanical complications. These findings are biologically and technically plausible and align with prior observations that complex lesion morphology increases procedural difficulty, wire manipulation, and device resistance. CTO recanalization often requires aggressive crossing strategies and prolonged instrumentation, increasing the likelihood of subintimal passage, vessel trauma, and dissection. Similarly, heavily calcified lesions limit vessel compliance, impair balloon expansion, and increase susceptibility to perforation or uncontrolled dissection during angioplasty or stent deployment. While many prior studies have linked CTOs and calcification primarily to long-term outcomes such as restenosis or target lesion revascularization, the present data emphasize their impact on immediate procedural safety. This distinction reinforces the need for careful pre-procedural planning, appropriate device selection, and consideration of adjunctive lesion preparation strategies in complex anatomy.

Beyond lesion characteristics, several procedural variables were associated in adjusted analyses with mechanical complications. The use of larger introducer sheaths (≥7F) and the need for multiple vascular access attempts were both linked to higher complication rates. These findings highlight the role of access strategy and procedural efficiency in mitigating mechanical risk. Multiple puncture attempts may reflect challenging access anatomy or suboptimal initial strategy and are likely to increase the risk of access site injury and procedural instability. Similarly, larger sheath sizes may exacerbate access-site-related trauma and limit maneuverability in tortuous vessels. Together, these results identify potentially modifiable procedural factors that may be targeted through operator training, access optimization, and careful procedural planning to improve peri-procedural safety. Notably, access route and stent type were not associated with mechanical complications after multivariable adjustment, suggesting that lesion complexity and procedural execution outweigh device choice in determining acute mechanical risk in this cohort.

From a clinical perspective, the findings of this study provide several practical insights. First, the low incidence of peri-procedural mechanical complications supports the overall safety of peripheral endovascular intervention in routine practice. Second, identification of high-risk anatomical and procedural features may aid operators in risk stratification, informed consent, and procedural planning. Finally, recognition of modifiable procedural factors reinforces the value of continuous quality improvement initiatives, particularly in medium-volume centers where procedural strategies and resource availability may differ from those of large tertiary referral centers. By focusing on peri-procedural mechanical complications rather than long-term clinical outcomes, this analysis complements existing literature, but given the low number of events, results should be interpreted as exploratory and hypothesis-generating.

### Limitations

This study has several limitations. First, it reflects the experience of a single center with a modest sample size accrued over a long inclusion period and may not be generalizable to institutions with different patient populations, operator expertise, or procedural strategies. Although the registry was prospectively maintained, the retrospective nature of the analysis introduces the potential for unmeasured confounding. Peri-procedural mechanical complications were recorded according to predefined categories within the institutional procedural database and were not externally adjudicated; however, the analysis was intentionally restricted to clearly defined mechanical events occurring during or immediately after the index intervention. Accordingly, reporting bias is possible, particularly for less clinically consequential angiographic findings (e.g., minor non-flow-limiting dissections) that may not require additional treatment and therefore may not be consistently recorded. In addition, the registry does not capture formal complication grading or treatment thresholds; thus, we could not stratify events by angiographic severity or clinical consequence. Lesion characteristics, including calcification severity, were assessed angiographically using qualitative criteria and may be subject to interobserver variability. Procedural techniques, devices, and operator experience evolved over the 10-year study period, and potential temporal effects were not explicitly modeled. The use of ultrasound guidance for vascular access also evolved over time and was heterogeneous across operators and study years; however, ultrasound-guided access was not captured as a dedicated registry variable and therefore could not be quantified or analyzed in relation to peri-procedural mechanical complications. In addition, the relatively low incidence of peri-procedural mechanical complications limited statistical power and constrained the complexity of multivariable modeling, potentially reducing the ability to detect weaker or interaction-level associations. Although most procedures were performed by four main operators, structured operator identifiers and operator-level volume metrics were not captured in the registry, precluding a formal assessment of operator volume–outcome associations. Finally, this analysis focused exclusively on peri-procedural mechanical complications and does not address long-term clinical outcomes, which are influenced by additional biological and therapeutic factors and warrant separate investigation.

## 5. Conclusions

In a real-world institutional registry spanning a decade of practice, peri-procedural mechanical complications during peripheral endovascular intervention were uncommon. Lesion complexity, particularly chronic total occlusion and severe calcification, and procedural factors such as sheath size and access difficulty were associated with increased mechanical risk in our adjusted analysis. These findings emphasize the importance of careful lesion assessment and procedural strategy in minimizing peri-procedural complications and provide actionable insights for quality improvement in routine endovascular practice.

## Figures and Tables

**Table 1:** Baseline Clinical and Lesion Characteristics.

Number of procedures analyzed	283
Variable	Value
Clinical Characteristics	
Age, years (mean ± SD)	67.5 ± 11.2
Male sex, n (%)	224 (79.2)
Hypertension, n (%)	195 (68.9)
Diabetes mellitus, n (%)	81 (28.6)
Dyslipidemia, n (%)	170 (60.1)
Active or former smoking, n (%)	199 (70.3)
Chronic kidney disease, n (%)	42 (14.8)
Coronary artery disease, n (%)	96 (33.9)
Lesion Characteristics	
Iliac segment treated, n (%)	135 (47.7)
Femoropopliteal segment treated, n (%)	145 (51.2)
Below-the-knee segment treated, n (%)	3 (1.1)
Chronic total occlusion, n (%)	108 (38.2)
Moderate-to-severe calcification, n (%)	138 (48.8)
TASC II class C/D, n (%)	119 (42.0)
Reference vessel diameter, mm (median [IQR])	6 [5–7]
Lesion length, mm (median [IQR])	80 [60–100]

Baseline demographic, clinical, and anatomical characteristics of the study population. Values are presented as mean ± standard deviation (SD) for continuous variables and number (percentage) for categorical variables. Lesion territories are not mutually exclusive; patients may have lesions involving more than one segment. As the analysis was performed at the index procedure level, lesion count (n = 283) represents the effective analytic sample.

**Table 2:** Procedural Characteristics.

Variable	Total Procedures Analyzed (n = 283)
Number of lesions treated per patient, median [IQR]	1 [1–1]
Access site—Femoral, n (%)	201 (71.0%)
Access site—Brachial, n (%)	16 (5.7%)
Access site—Radial, n (%)	66 (23.3%)
Introducer sheath size ≥7F, n (%)	18 (6.4%)
≥3 Access attempts, n (%)	70 (24.7%)
Pre-dilatation performed, n (%)	161 (56.9)
Post-dilatation performed, n (%)	137 (48.4)
Direct stenting, n (%)	109 (38.5)
Stent type—Bare-metal, n (%)	177 (62.4)
Stent type—Drug-eluting, n (%)	71 (25.0)
Stent type—Covered, n (%)	35 (12.6)
Procedural success (lesion revascularization completed), n (%)	279 (98.6)

Values are presented as numbers (percentage). Variables include access route, introducer sheath size, number of access attempts, balloon pre- and post-dilatation, and type of stents used.

**Table 3:** Peri-Procedural Mechanical Complications (Primary Endpoint).

Category	n (%)
**Total Peri-Procedural Events**	283
**Type**	
Any peri-procedural mechanical complication	9 (3.2)
Arterial dissection	6 (2.1)
Arterial perforation	1 (0.4)
Other mechanical complication	2 (0.7)
Peri-procedural bleeding	0 (0.0)

Peri-procedural mechanical complications were defined as mechanical adverse events occurring during or immediately after the index endovascular intervention and directly related to catheter manipulation, device deployment, or vascular access. Percentages are calculated using the total number of index procedures as the denominator. More than one complication could occur during a single procedure. Events were operator-reported in predefined registry categories without external adjudication or formal angiographic grading. Dissections represent operator-documented peri-procedural dissections; trivial, non-flow-limiting dissections not recorded by the operator may not be captured. The ‘other mechanical complication’ category comprised stent delivery failure (n = 1) and popliteal breach with calf hematoma (n = 1).

**Table 4:** Multivariable Predictors of Peri-Procedural Mechanical Complications During Peripheral Endovascular Interventions.

Predictor	Adjusted OR	95% CI	*p* Value
**Chronic total occlusion**	1.89	1.14–3.11	0.014
**Moderate-to-severe calcification**	1.74	1.03–2.93	0.039
**Introducer sheath ≥7F**	2.08	1.21–3.57	0.007
**≥3 vascular access attempts**	1.67	1.00–2.81	0.048
**Lesion location (iliac vs. femoropopliteal)**	1.12	0.65–1.94	0.68
**Access route (femoral vs. others)**	1.05	0.62–1.76	0.84
**Stent type (bare metal vs. others)**	0.94	0.56–1.60	0.82

Odds ratios are adjusted for all variables included in the multivariable logistic regression model.

## Data Availability

The original contributions presented in the study are included in the article, further inquiries can be directed to the corresponding author. (specify the reason for the restriction).
